# Mobilisation of critically ill patients receiving norepinephrine: a retrospective cohort study

**DOI:** 10.1186/s13054-022-04245-0

**Published:** 2022-11-25

**Authors:** Maximilian Lindholz, Clara M. Schellenberg, Julius J. Grunow, Simone Kagerbauer, Annette Milnik, Daniel Zickler, Stefan Angermair, Anett Reißhauer, Martin Witzenrath, Mario Menk, Sebastian Boie, Felix Balzer, Stefan J. Schaller

**Affiliations:** 1 Department of Anesthesiology and Operative Intensive Care Medicine (CVK, CCM), Charité – Universitätsmedizin Berlin, Corporate Member of Freie Universität Berlin and Humboldt-Universität Zu Berlin, Berlin, Germany; 2 Department of Anesthesiology and Intensive Care, School of Medicine, Technical University of Munich, Munich, Germany; 3 Department of Anesthesiology and Intensive Care Medicine, Ulm University, Ulm, Germany; 4 Division of Molecular Neuroscience, University of Basel, Basel, Switzerland; 5 Department of Nephrology and Medical Intensive Care, Charité – Universitätsmedizin Berlin, Corporate Member of Freie Universität Berlin and Humboldt-Universität Zu Berlin, Berlin, Germany; 6 Department of Anesthesiology and Operative Intensive Care Medicine (CBF), Charité – Universitätsmedizin Berlin, Corporate Member of Freie Universität Berlin and Humboldt-Universität Zu Berlin, Berlin, Germany; 7 Department of Physical Medicine, Charité – Universitätsmedizin Berlin, Corporate Member of Freie Universität Berlin and Humboldt-Universität Zu Berlin, Berlin, Germany; 8 Department of Infectious Diseases and Pulmonary Medicine, Charité – Universitätsmedizin Berlin, Corporate Member of Freie Universität Berlin and Humboldt-Universität Zu Berlin, Berlin, Germany; 9 Institute of Medical Informatics, Charité – Universitätsmedizin Berlin, Corporate Member of Freie Universität Berlin and Humboldt-Universität Zu Berlin, Berlin, Germany

**Keywords:** Early mobilisation, Early ambulation, Norepinephrine, Adverse events, Supervised machine learning

## Abstract

**Background:**

Mobilisation and exercise intervention in general are safe and feasible in critically ill patients. For patients requiring catecholamines, however, doses of norepinephrine safe for mobilisation in the intensive care unit (ICU) are not defined. This study aimed to describe mobilisation practice in our hospital and identify doses of norepinephrine that allowed a safe mobilisation.

**Methods:**

We conducted a retrospective single-centre cohort study of 16 ICUs at a university hospital in Germany with patients admitted between March 2018 and November 2021. Data were collected from our patient data management system. We analysed the effect of norepinephrine on level (ICU Mobility Scale) and frequency (units per day) of mobilisation, early mobilisation (within 72 h of ICU admission), mortality, and rate of adverse events. Data were extracted from free-text mobilisation entries using supervised machine learning (support vector machine). Statistical analyses were done using (generalised) linear (mixed-effect) models, as well as chi-square tests and ANOVAs.

**Results:**

A total of 12,462 patients were analysed in this study. They received a total of 59,415 mobilisation units. Of these patients, 842 (6.8%) received mobilisation under continuous norepinephrine administration. Norepinephrine administration was negatively associated with the frequency of mobilisation (adjusted difference -0.07 mobilisations per day; 95% CI − 0.09, − 0.05; *p* ≤ 0.001) and early mobilisation (adjusted OR 0.83; 95% CI 0.76, 0.90; *p* ≤ 0.001), while a higher norepinephrine dose corresponded to a lower chance to be mobilised out-of-bed (adjusted OR 0.01; 95% CI 0.00, 0.04;* p* ≤ 0.001). Mobilisation with norepinephrine did not significantly affect mortality (*p* > 0.1). Higher compared to lower doses of norepinephrine did not lead to a significant increase in adverse events in our practice (*p* > 0.1). We identified that mobilisation was safe with up to 0.20 µg/kg/min norepinephrine for out-of-bed (IMS ≥ 2) and 0.33 µg/kg/min for in-bed (IMS 0–1) mobilisation.

**Conclusions:**

Mobilisation with norepinephrine can be done safely when considering the status of the patient and safety guidelines. We demonstrated that safe mobilisation was possible with norepinephrine doses up to 0.20 µg/kg/min for out-of-bed (IMS ≥ 2) and 0.33 µg/kg/min for in-bed (IMS 0–1) mobilisation.

**Supplementary Information:**

The online version contains supplementary material available at 10.1186/s13054-022-04245-0.

## Introduction

Vasoactive drugs are essential in managing vasodilatory shock, not responsive to volume therapy [1, 2]. They are also administered in cardiogenic and hypovolemic shock [3]. Vasopressors generally bind to specific (e.g. adrenergic) receptors, thereby creating vasoconstriction and raising the mean arterial pressure (MAP) [2]. Sustaining an adequate MAP is crucial to organ perfusion; however, vasopressors also cause adverse effects, including tachycardia and arrhythmias [1, 2]. For most clinical conditions requiring vasoactive drugs, norepinephrine is the first-line therapy [4].

Vasopressors are an indicator of hemodynamic instability [3] and are perceived as a barrier to starting early mobilisation [5, 6], resulting in less mobilisation [4]. While sufficient mobilisation of patients is a crucial step in rehabilitation in the intensive care unit (ICU) [6–10], too much mobilisation, especially active forms, inherits the potential of harm [11].

There is little to no expert consensus on safe doses of vasoactive drugs in the mobilisation of the critically ill, as well as a lack of quantitative data [12–14]. A systematic review of safety criteria in early mobilisation in 2017 stated that there was no reported safe dose of vasoactive drugs up to which mobilisation can be recommended [14]. A recent systematic review came to the same conclusion and pointed out that data concerning a safe dose of norepinephrine for early mobilisation is inconclusive [13].

This report focuses on norepinephrine since it is the most often used vasopressor [2]. Consequently, this study aims to identify the impact of norepinephrine on mobilisation in general and in a dose-specific manner.

## Methods

### Study design and data sources

Patient data were extracted from the Charité—Universitätsmedizin Berlin ICU patient data management system (PDMS) to conduct a single-centre retrospective cohort study. Charité extends over different campuses: Campus Charité Mitte (CCM), Campus Virchow-Klinikum (CVK) and Campus Benjamin Franklin (CBF). We included data from 16 intensive care units from March 2018 to November 2021 from the following departments: Department of Anesthesiology and Operative Intensive Care Medicine (CVK, CCM), Department of Anesthesiology and Operative Intensive Care Medicine (CBF), Department of Nephrology and Medical Intensive Care, and Department of Infectious Diseases and Pulmonary Medicine. The analysed database included the following patient details: age; sex; body height and weight; hospital admission and discharge details, name of the ICU(s); daily records of drug administration, drug dosage; diagnoses, comorbidities, and treatment details as documented in the ICD-10 and ICD-OPS Scores; Richmond Agitation-Sedation Scale (RASS) [15], Acute Physiology and Chronic Health Evaluation II (APACHE II) [16], Sequential Organ Failure Assessment score (SOFA Score) [17] as well as free-text mobilisation entries.

This analysis is covered by the approval EA4/084/21 of the ethics committee of the Charité—Universitätsmedizin Berlin from 30.03.2022. According to German regulations, the ethics committee waived the necessity of informed consent due to the retrospective analysis of routine clinical data.

### Patient selection

Data were available from 17,913 patients admitted to an ICU in the study period and could uniquely be identified. We excluded patients under 18 years and patients that stayed less than 24 h, as well as patients whose data on age, sex, RASS, or SOFA Scores were missing.

### Norepinephrine

To investigate the general impact of norepinephrine on mobilisation practice, patients were classified if they received norepinephrine during their ICU stay at any time (yes/no).

We used the continuous rate given (dosing unit µg/kg/min) for the dose-specific analysis during mobilisation. For the safety analysis, we additionally identified if norepinephrine was applied during a mobilisation unit (yes/no).

We considered doses of > 0.2 µg/kg/min as high doses of norepinephrine and between 0.05 and 0.2 µg/kg/min as moderate, below 0.05 as low, comparable to previous research [13, 18]. Mobilisation units were classified accordingly (mobilisation under high/moderate/low norepinephrine).

### Covariates and confounder

We selected a priori the following covariates and confounders for risk adjustment and to correct for potential bias based on clinical experience and data availability. Patient-level covariates were age (years), sex (male/female), obesity (ICD-10-Code E66*), COVID-19 (manually extracted from discharge letter as relevant or main diagnosis and positive PCR test or based on ICD-10 Code U07.1), APACHE II and SOFA Score (organ dysfunction) at ICU admission, Elixhauser Comorbidities Index at discharge (labelled according to Quan [19]) and the RASS.

The following therapy details during the stay in the ICU were also used as patient-level covariates: dialysis, ECMO, high flow nasal cannula, non-invasive ventilation, intubation together with invasive mechanical ventilation, and tracheostomy at any time point (yes/no for each variable).

On a hospital level, we used the type of ICU as a potential confounder. If a patient was treated in different types of ICUs (e.g. medical and neurocritical ICU), we used the category “Multiple ICU types”.

For 2,499 cases, we did not have an admission APACHE II score; therefore, we imputed with the mean of the other 9,963 patients (mean = 20.04).

### ICU Mobility Scale (IMS)

The IMS is an 11-point ordinal scale that assesses the intensity and milestones of mobility in critically ill patients. It has strong interrater reliability and validity [20, 21]. To generate IMS score for each mobilisation unit we extracted free-text mobilisation entries from the mobilisation documentation (typically done by nurses or physical therapists) in our PDMS. Staff members used to some extent individually generated templates for the free-text entries.

IMS scores were generated from the free-text mobilisation entries using a supervised machine learning approach. We were not able to differentiate between a score of 7 or 8 because there is insufficient information on the number of critical care team members assisting in mobilisation in our routine documentation. Although the assistance is mostly carried out by one person, we took a conservative approach and assigned an IMS of 7 in those cases. The highest achieved IMS level of one mobilisation session was used as described [11, 22].

The training and test data were generated by manual labelling 0.8% of the available data: out of a total of 158,272 free-text entries, we randomly selected and blinded 1235 entries; two raters independently labelled these entries with the IMS and additionally identified data, that was wrongly documented (labelled as non-mobilisation entries). We then checked for discrepancies between the ratings of the two raters (5 out of 1235 entries; 0.4%) and decided together on these entries.

The 1235 manually labelled entries were then randomly split into 80% training data and 20% test data. The training data were supplied to a support vector machine (SVM) model and a Naïve Bayes (NB) classifier by using the quanteda R package [23].

Based on the test data, the SVM got an accuracy of 0.91 (95% confidence interval (CI) 0.87–0.94) and outperformed the NB classifier (accuracy 0.67 (95% CI 0.64–0.71)). The Cohen’s kappa of the SVM is 0.89. The “No Information Rate” is 36%, meaning that if you choose the majority class, you will be right at 36%. This indicates that a model with 91% outperforms chance. We used the SVM model to generate the IMS scores for all 158,272 free-text entries. We then removed 17,551 entries labelled as non-mobilisation entries, leaving us with 140,721 IMS scores.

Afterwards, we used regular expressions to correct for further commonly found mislabels that were not already labelled as non-mobilisation entries by the SVM, resulting in the removal of an additional 3,686 entries and leaving us with a final set of 137,035 mobilisations with IMS scores. 61,422 of these entries took place while the patients were admitted to an ICU and in our study period. As a final quality check, we randomly selected 200 of these SVM-labelled mobilisation entries, blinded them, and again manually labelled them with two independent raters. The concordance rate of the manually labelled and SVM-labelled scores was on average 92%.

The SVM-based automated text-labelling code is available online at github.com [24].

Clinical recommendations distinguish between the two categories of in-bed and out-of-bed mobilisation. For each individual mobilisation unit, we used the SVM-labelled IMS scores and classified them into in-bed or out-of-bed mobilisation. However, there are different definitions in the literature as to which IMS level can be considered in-bed and out-of-bed, with options ranging from IMS 0–1 to IMS 0–3 as in-bed mobilisation [12, 25, 26]. Therefore, we chose the most conservative option (IMS 0–1) as in-bed mobilisation but added a sensitivity analysis using individual IMS levels up to IMS 4.

### Adverse events

Adverse events were manually extracted from the free-text entries and PDMS during the mobilisation units under norepinephrine, independently by two raters. The agreement between the raters was 100%.

Adverse events (AE) were defined as any unfavourable symptoms or events associated with a mobilisation session, such as mobilisation-induced physiological changes (e.g. hypo- and hypertension, brady-/tachycardia), respiratory symptoms (e.g. dyspnoea, desaturation below 90%), agitation or further minor adverse events like dizziness. Severe adverse events were defined as adverse events that are life-threatening, deadly or lead to permanent disability. These would comprise, for example, cardiac arrest, loss of consciousness and death [8, 13].

### Outcome

Primary outcomes on mobilisation were:

1. Frequency: average number of mobilisations per day
2. Early mobilisation (i.e. mobilisation in the first 72 h [8, 27, 28]): yes/no
3. Intensity of mobilisation: in-bed (IMS 0–1)/out-of-bed (IMS 2–10)

Secondary outcomes on safety were:

1. Adverse event when mobilised under norepinephrine: yes/no
2. Hospital mortality: yes/no

### Statistical analysis

Analyses were done in R (R version 4.1.1 (2021-08-10). Significance testing for group differences was done with chi-square tests for categorical data and Wilcoxon signed-rank test for continuous data using the tableone package [29].

For the main analyses, we applied multivariate linear and mixed models in combination with type III ANOVA using the car package [30] and lme4 package [31].

The effect of norepinephrine (yes/no) on mobilisation frequency was analysed using a general linear model. The effect of norepinephrine (yes/no) on early mobilisation and hospital mortality was analysed by using generalised linear models with a binomial distribution.

For the dose-specific analysis, we assessed the effect of the norepinephrine rate (in µg/kg/min) on the level of mobilisation by using a generalised mixed model with a binomial distribution and subject as a random effect.

To get an unbiased mean and 95th percentile of norepinephrine doses for in-bed and out-of-bed mobilisation, as well as the sensitivity analysis using individual IMS levels till IMS 4, we used the average duration per mobilisation unit and level for each patient. We reported the 95th percentile rather than the maximum value to account for extreme outliers to reduce bias.

To assess the safety of mobilisation under continuous norepinephrine administration, we compared the hospital mortality of the group of patients mobilised during norepinephrine application to those not mobilised during norepinephrine application by using a generalised linear model with a binomial distribution.

Age, sex, obesity, admission Apache-II, admission SOFA Scores, Elixhauser Comorbidities Index, COVID-19, therapy details (ECMO, high flow nasal cannula, non-invasive ventilation, intubation together with invasive mechanical ventilation, tracheostomy) and type of ICU were included as covariates in all models, except the adverse event models, which were unadjusted. In the generalised mixed model, we corrected for the highest absolute RASS on a given day before the mobilisation; in all other models, we included the median absolute RASS. The median absolute score is calculated from daily averages.

Uncorrected estimates are based on the same models without adding any covariates.

We assessed the influence of norepinephrine dosage and type of mobilisation (in-bed vs. out-of-bed) on the occurrence (yes/no) of adverse events using generalised linear models with and without interaction terms. As a sensitivity analysis, we also analysed the effect of dose and individual IMS on the occurrence of adverse events using the same approach. For the individual IMS analysis, we collapsed the data from IMS 5–10 due to small individual sample sizes in the higher IMS groups and used IMS as a linear predictor. Because of the small number of adverse events, we used a Monte Carlo procedure [32] to calculate empirical *p* values.

A nominal alpha level of 0.05 was considered statistically significant.

## Results

**Fig. 1:**
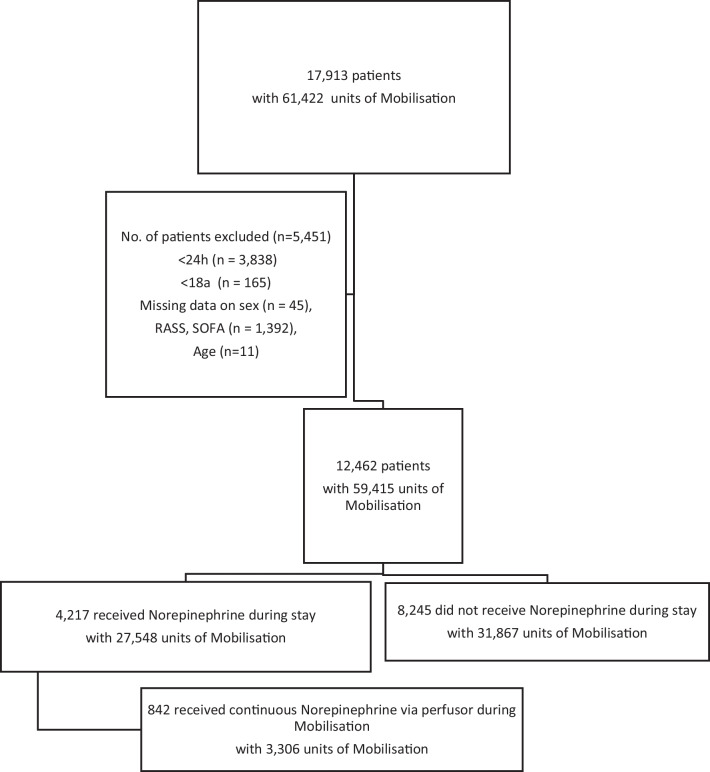
Flow diagram of available data, exclusion reasons and missing data

A total of 12,462 patients were selected as eligible. These patients received a total number of 59,415 units of mobilisation; 53% of the mobilisation units took place out-of-bed and 47% took place in-bed; 4217 patients (34%) received norepinephrine during their stay, and 8245 (66%) did not receive norepinephrine. Of the 4217 patients, 842 received mobilisation during continuous norepinephrine administration (20% of norepinephrine receiving patients), with a total of 3306 therapy units (12% of therapy units in this group) (Fig. 1).

Patients receiving norepinephrine compared to patients not receiving norepinephrine differed in all included covariates and confounders except for sex and age (Table 1). For differences in comorbidities and diagnosis at admission between the two groups, see Additional file 1: Table S1 and S2.

**Table 1:** Baseline characteristics of patients who received norepinephrine and those who did not receive norepinephrine during their stay

	No norepinephrine	Norepinephrine	*p* value
*n*	8245	4217	
Age	66.0 [54.0, 77.0]	67.0 [56.0, 77.0]	0.213
Elixhauser comorbidity index [19]	0.0 [0.0, 11.0]	4.0 [0.0, 15.0]	< 0.001
Admission APACHE II	20.0 [13.0, 23.0]	20.0 [18.0, 28.0]	< 0.001
Admission SOFA	4.0 [2.0, 7.0]	6.0 [3.0, 9.0]	< 0.001
Median absolute RASS	0.5 [0.0, 1.0]	1.0 [0.0, 2.0]	< 0.001
Female sex	3380 (41.0)	1693 (40.1)	0.37
Obesity (ICD-10)	190 (2.3)	132 (3.1)	0.007
Dialysis	958 (11.6)	760 (18.0)	< 0.001
ECMO	113 (1.4)	122 (2.9)	< 0.001
Highflow	1830 (22.2)	1079 (25.6)	< 0.001
Intubated	1504 (18.2)	1390 (33.0)	< 0.001
NIV	2710 (32.9)	1822 (43.2)	< 0.001
Tracheostomy (%)	316 (3.8)	345 (8.2)	< 0.001
Type of ICU (%)			< 0.001
Cardiac surgery	134 (1.6)	814 (19.3)	
Interdisciplinary operative	4096 (49.7)	2124 (50.4)	
Internal medicine	2882 (35.0)	413 (9.8)	
Multiple specialties	419 (5.1)	460 (10.9)	
Neurocritical	714 (8.7)	406 (9.6)	
COVID-19 (%)	496 (6.0)	293 (6.9)	0.047

Data are presented as median [IQR] or *n* (%)

*APACHE II* Acute Physiology and Chronic Health Evaluation II, *ICU* Intensive care unit, *ICD* International Statistical Classification of Diseases and Related Health Problems, *COVID-19* coronavirus disease 2019, *NIV* non-invasive ventilation

### General impact of norepinephrine on mobilisation

People who received norepinephrine were less likely to be mobilised than to those not receiving norepinephrine (adjusted difference − 0.07 mobilisations per day; 95% CI − 0.09, − 0.05; *p* ≤ 0.001). They were also less likely to receive early mobilisation (adjusted OR 0.83; 95% CI 0.76, 0.90; *p* ≤ 0.001).

There was no significant difference in hospital mortality between people receiving norepinephrine and those not receiving norepinephrine during their ICU stay (adjusted OR 1.04; 95% CI 0.91, 1.19; *p* = 0.58, see Table 2).

**Table 2:** General impact of norepinephrine on mobilisation, comparing patients who received norepinephrine and those who did not receive norepinephrine during their stay

	No norepinephrine	Norepinephrine	Unadjusted difference (95% CI)	Adjusted difference
	(*n* = 8245)	(*n* = 4217)		
Number of mobilisations per day (mean (SD))	0.38 (0.5)	0.35 (0.4)	− 0.03 (− 0.05, − 0.01)	− 0.07 (− 0.09, − 0.05)
Early mobilisation (*n* (%))	3323 (40.3)	1645 (39.0)	OR 0.95 (0.88, 1.02)	OR 0.83 (0.76, 0.90)
Hospital mortality (*n* (%))	1607 (19.5)	1011 (24.0)	OR 1.30 (1.19, 1.42)	OR 1.04 (0.91, 1.19)

*CI* confidence interval, *SD* standard deviation

### Dose-specific impact of norepinephrine on mobilisation

For 842 patients, 3306 mobilisation units were done with continuous norepinephrine administration; 2397 were in-bed mobilisations (IMS 0–1; *n* = 638 patients) and 909 out-of-bed mobilisations (IMS ≥ 2; *n* = 395 patients). There were 191 patients, who received both in-bed and out-of-bed mobilisation during their stay.

The mean norepinephrine rate for in-bed mobilisation was 0.12 (SD 0.11) µg/kg/min and for out-of-bed mobilisation 0.07 (SD 0.06) µg/kg/min. The 95th percentile was 0.33 µg/kg/min for in-bed (IMS 0–1) mobilisation and 0.20 µg/kg/min for out-of-bed (IMS ≥ 2) mobilisation. The results of the sensitivity analysis are presented in Table 3 with similar results.

**Table 3:** Sensitivity analysis of norepinephrine dose in individual IMS levels with IMS 5–10 combined, because of small sample sizes

IMS	NA dose mean (SD)	NA dose upper 95th percentile	Patients *n*	AEs *n* (%)
0	0.12 (0.10)	0.32	614	20 (3.3)
1	0.11 (0.10)	0.35	63	0 (0.0)
2	0.07 (0.07)	0.18	89	2 (2.3)
3	0.08 (0.07)	0.20	223	16 (7.2)
4	0.07 (0.06)	0.20	86	5 (5.8)
5–10	0.07 (0.06)	0.16	132	4 (3.0)

**Fig. 2:**
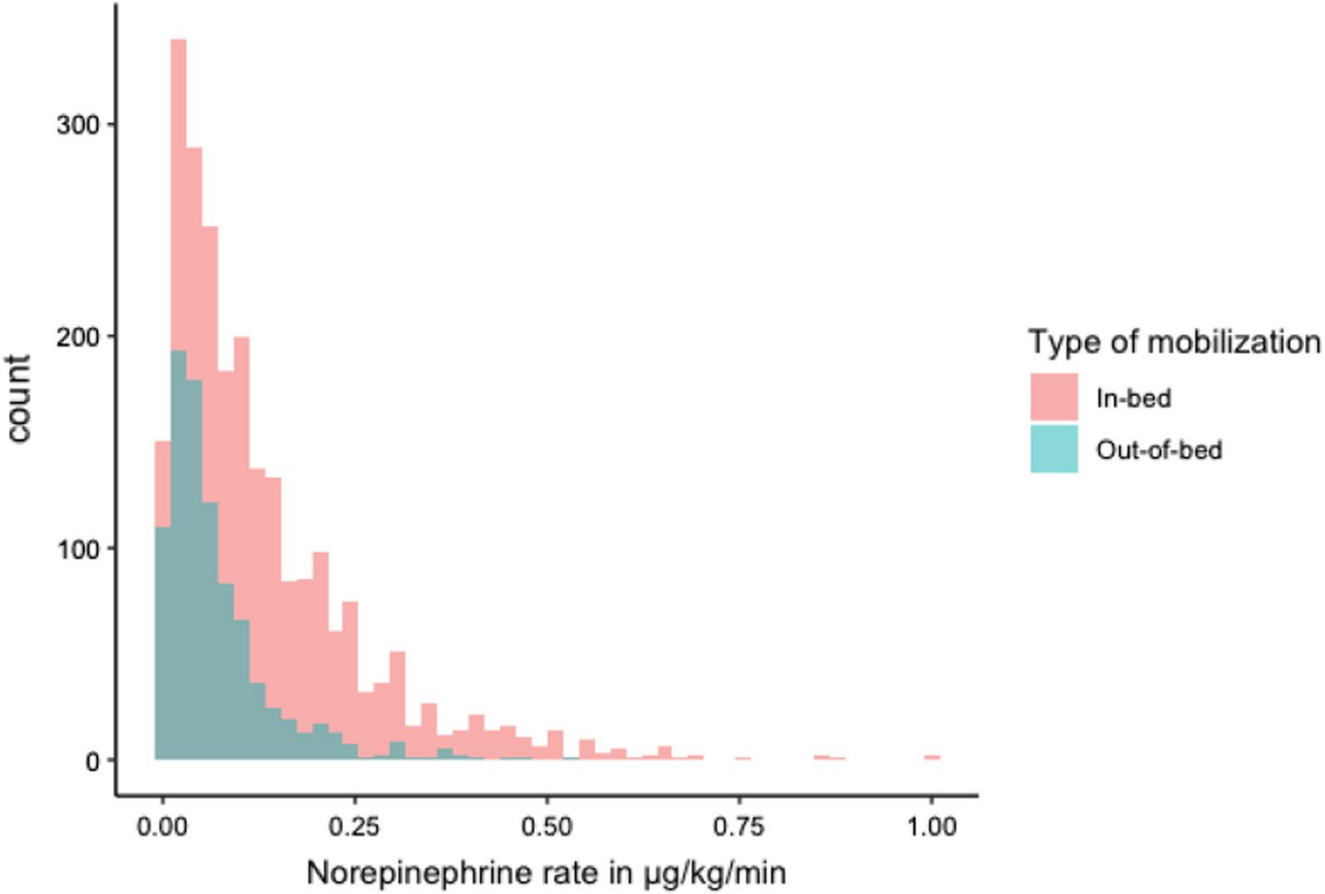
Histogram of norepinephrine rate (mcg/kg/min) during mobilisation for in-bed (IMS 0–1) and out-of-bed mobilisation (IMS ≥ 2)

A higher norepinephrine rate was associated with a reduced chance for patients to be mobilised out of bed (adjusted OR 0.009; 95% CI 0.002, 0.040; *p* ≤ 0.001; see Fig. 2).

Out of the 3306 mobilisation units, 16% (n = 525) were done with high-dose norepinephrine (> 0.2 µg/kg/min; Table 3), with most of them (90%) being in-bed mobilisation. Moderate doses were administered in 46% of cases (n = 1529), with 75% being in-bed mobilisation. Low doses accounted for 38% of cases (n = 1252), with 62% being in-bed mobilisations (see Table 4). In patients without continuous norepinephrine administration, the rate of in-bed-mobilisation was 45%.

**Table 4:** Number and percentage of mobilisation units under high/moderate/low norepinephrine, shown separately for in-bed and out-of-bed mobilisation

Norepinephrine	Out-of-bed mobilisation	In-bed mobilisation	All mobilisations
High dose	52 (10%)	473 (90%)	525 (100%)
Moderate dose	380 (25%)	1149 (75%)	1529 (100%)
Low dose	477 (38%)	775 (62%)	1252 (100%)
Total	909 (27%)	2397 (73%)	3306 (100%)

Mortality was not significantly different between patients with and without norepinephrine during mobilisation (adjusted OR 0.96; 95% CI 0.77, 1.19; *p* = 0.68).

There were a total of 47 adverse events (1%). There was no significant effect of norepinephrine dose on the rate of adverse events (OR 0.2; 95% CI 0.0, 3.8; *p* > 0.1). There was no significant interaction between dose and group (in-bed, out-of-bed) on the rate of adverse events (*p* > 0.1). However, there were significantly more adverse events in the group of out-of-bed-mobilisation, regardless of the norepinephrine dose (OR 3.3; 95% CI 1.8, 6.1; *p* = 0.001, see Table 5).

**Table 5:** Number and percentage of adverse events under high/moderate/low norepinephrine, shown separately for in-bed and out-of-bed mobilisation

	Adverse event	No adverse event	Total
High-dose			
Out-of-bed mobilisation	2 (4%)	50 (96%)	52 (100%)
In-bed mobilisation	2 (< 1%)	471 (> 99%)	473 (100%)
Moderate-dose			
Out-of-bed mobilisation	11 (3%)	369 (97%)	380 (100%)
In-bed mobilisation	11 (1%)	1138 (99%)	1149 (100%)
Low-dose			
Out-of-bed mobilisation	14 (3%)	463 (967	477 (100%)
In-bed mobilisation	7 (1%)	768 (99%)	775(100%)
Total	47 (1%)	3259 (99%)	3306 (100%)

The adverse events were cardiovascular (hypo-/hypertension, or tachycardia, *n* = 30), respiratory (e.g. dyspnoea, desaturation below 90% (*n* = 5)), agitation (*n* = 3), as well as different minor adverse events (e.g. dizziness) (*n* = 8). We also registered one severe adverse event in 3306 mobilisation sessions under norepinephrine. The event resulted in the need to resuscitate the patient; resuscitation was successful. The mobilisation unit was an out-of-bed mobilisation with a moderate norepinephrine dosage (0.14 mcg/kg/min).

In our sensitivity analysis on the rate of adverse events using individual IMS levels up to IMS 4, instead of in-bed versus out-of-bed, we confirmed a missing effect of norepinephrine rate on the occurrence of adverse events (OR: 0.1; 95% CI 0.0, 2.8; *p* > 0.1). Furthermore, there was no significant interaction between IMS level and norepinephrine dose on the rate of adverse events (*p* > 0.1). However, there were significantly more adverse events at higher IMS levels (OR 1.3; 95% CI 1.1, 1.5; *p* < 0.001, see Tables 3 and 5).

## Discussion

The present study explored the effect of norepinephrine on the mobilisation and safety of these mobilisations. Patients receiving norepinephrine were less likely to be mobilised early, were mobilised less often and were mobilised mostly in-bed only.

The delay in initiation of mobilisation and the reduced frequency of mobilisation is not surprising, with hemodynamic instability being one of the main barriers to the mobilisation of critically ill patients [5, 11, 14]. Higher norepinephrine dosages were associated with decreased mobilisation level for patients who received a continuous norepinephrine application. Mobilisation during norepinephrine administration was predominantly (73%) in-bed, while a minority (45%) of mobilisation was in-bed without noradrenaline administration. The important difference between these mobilisation forms is the verticalisation of the patients. These postural changes are accompanied by haemodynamic changes and strain on the blood pressure regulation of the patients. As an increase in achieved IMS levels also leads to more strain on the circulatory system, an increase in adverse events (mainly being hypotension) would be physiologically understandable [1, 3, 4, 20, 33, 34].

A recent systematic review on early mobilisation with vasoactive drugs found inconsistent recommendations on early mobilisation with vasoactive drugs. The review reported no serious adverse events associated with early mobilisation; adverse events were mainly transient physiological changes (e.g. hypotension) [13]. In our larger cohort, we recorded one severe adverse event; which is low compared to the largest available randomised data available on active forms of mobilisation only [11]. Other than that, we mainly found transient physiological changes (mainly hypotension). There is, to date, no clinical consensus on safe doses of norepinephrine up to which mobilisation can take place [12–14].

Furthermore, there is hardly any quantitative data on adverse events during mobilisation under norepinephrine. A study by Hickmann et al. analysed the mobilisation practices in their hospital, intending to demonstrate that early mobilisation is feasible despite the need for mechanical ventilation, renal replacement therapy, high FiO_2_ and vasopressor usage. Their study described 361 mobilisation units with a mean dose of 0.10 µg/kg/min norepinephrine, 19 of which were done with doses of > 0.2 µg/kg/min. They reported no severe adverse event and found only transient adverse events in patients mobilised during vasopressors administration [35]. Another way of assessing doses of norepinephrine considered safe is to look at study inclusion criteria. These criteria are based on clinical reasoning rather than quantitative data. Two studies by McWilliams et al. considered doses of > 0.2 µg/kg/min to be a contraindication for mobilisation [36, 37]. A study by Sommers et al. considered doses of ≥ 0.1 µg/kg/min to be a contraindication, as defined in their study protocol [38]. It is essential to understand that Sommers et al. investigated a treadmill system, i.e. out-of-bed active mobilisation only; therefore, the more conservative approach seems reasonable. The largest international mobilisation trial (TEAM RCT) used a combined noradrenaline/adrenaline infusion rate of ≤ 0.2 µg/kg/min, or if there was an increase by more than 25% in the last 6 h, the dose had to be < 0.1 µg/kg/min [22].

Given that in our large cohort, there was no increase in adverse events with a higher dosage, we conclude that mobilisation during norepinephrine administration can be safe. Furthermore, the mortality did not significantly differ in the group of people receiving norepinephrine during mobilisation or not and between people receiving norepinephrine at any time point or not. However, one serious adverse event indicates that mobilisation in critically ill patients needs to be done with care.

Based on our data, we would recommend the 95th percentiles of our observations as dosages up to which safe mobilisation can take place; we recommend 0.2 µg/kg/min norepinephrine for out-of-bed (IMS >  = 2) and 0.33 µg/kg/min norepinephrine for in-bed (IMS 0–1) mobilisation as proposed dosage thresholds. Our out-of-bed threshold is identical to Yang et al., who developed a safety protocol for mobilisation based on a systematic literature review. They considered norepinephrine doses smaller than 0.2 µg/kg/min and no increase in the past 2 h as a safety criterion for out-of-bed mobilisation [39]. We did not find comparable recommendations for in-bed mobilisation.

### Study limitations

The study is a single-centre retrospective cohort study. However, Charité – Universitätsmedizin Berlin is the largest university hospital in Europe, with three campuses, and we incorporated data from 16 ICUs of different departments. Information on patients was extracted from routine clinical data. We excluded patients with missing data on our control covariates, except for patients with missing admission APACHE II scores, which we imputed. The IMS in general shows a high interrater reliability [20]. Our supervised machine learning approach also yielded very high accuracy, probably due to semi-structured free-text mobilisation entries; direct documentation of the IMS after each session might still outperform our approach. Nonetheless, given our large data set and data availability, using a supervised machine learning approach was the most feasible option. Since (serious) adverse events were assessed manually during all mobilisations, the assessment was only feasible if norepinephrine was applied, limiting a comparison with a control group without norepinephrine.

## Conclusions

Our retrospective study demonstrated that patients receiving norepinephrine during their ICU stay received a lower frequency of mobilisation (mobilisations per day) and less early mobilisation. Higher norepinephrine rates administered during mobilisation were negatively correlated with the level of mobilisation. There was no increase in adverse events with higher norepinephrine doses; however, the rate of adverse events increased with higher IMS levels; this effect was independent of the norepinephrine rate administered. The mortality did not significantly increase for people who received mobilisation under norepinephrine. It can be assumed that doses up to 0.20 mcg/kg/min for out-of-bed mobilisation (IMS ≥ 2) and doses of up to 0.33 mcg/kg/min for in-bed (IMS 0–1) mobilisation seem to be safe.

## Supplementary Information

**Additional file 1**. **Table S1.** Individual elements of the Elixhauser index. **Table S2.** All Admission diagnoses with n > 50.

## Data Availability

The data analysed are not publicly available due to German privacy regulations. Given a reasonable scientific request, the corresponding author can make the data available via a collaboration agreement.

## Abbreviations

AE: Adverse event
APACHE II: Acute Physiology and Chronic Health Evaluation II
CBF: Campus Benjamin Franklin
CCM: Campus Charité Mitte
CVK: Campus Virchow-Klinikum
ICU: Intensive care unit
IMS: ICU Mobility Scale
MAP: Mean arterial pressure
NB: Naïve Bayes
PDMS: Patient data management system
RASS: Richmond Agitation-Sedation Scale
SOFA Score: Sequential Organ Failure Assessment Score
SVM: Support vector machine
