# Supplementary material for: Mobilisation of critically ill patients receiving norepinephrine: a retrospective cohort study
Source: Crit Care. 2022 Nov 25;26:362. doi: 10.1186/s13054-022-04245-0 (PMC9700948; doi:10.1186/s13054-022-04245-0)
Supplement: Supplementary file 1 — Additional file 1. Table S1. Individual elements of the Elixhauser index. Table S2. All Admission diagnoses with n > 50. [file 13054_2022_4245_MOESM1_ESM.pdf]

Electronic supplementary material (ESM) – Additional File 1 Mobilisation of critically ill patients receiving Norepinephrine. A retrospective cohort study.

Maximilian Lindholz, Clara M Schellenberg, Julius J Grunow, Simone Kagerbauer, Annette Milnik, Daniel Zickler, Stefan Angermair, Anett Reißhauer, Martin Witzenrath, Mario Menk, Sebastian Boie, Felix Balzer, Dr. Stefan J Schaller

Content

TABLE S1. 2

TABLE S2. 3

Table S1. Individual elements of the Elixhauser index.

	No norpepinephrine	Norepinephrine	p-value
**n**	8245	4217	
**Chronic heart failure**	523 (6.3)	418 (9.9)	<0.001
**Cardiac arrythmias**	885 (10.7)	683 (16.2)	<0.001
**Valvular disease**	262 (3.2)	438 (10.4)	<0.001
**Pulmonary circulation disorders**	217 (2.6)	147 (3.5)	0.009
**Peripheral vascular disorders**	355 (4.3)	321 (7.6)	<0.001
**Hypertension**	1360 (16.5)	983 (23.3)	<0.001
**Hypertension with chronic complications**	184 (2.2)	208 (4.9)	<0.001
**Paralyisis**	306 (3.7)	174 (4.1)	0.276
**Neurodegenerative disorders**	434 (5.3)	226 (5.4)	0.855
**Chronic pulmonary disease**	433 (5.3)	229 (5.4)	0.705
**Diabetes**	662 (8.0)	524 (12.4)	<0.001
**Diabetes with chronic complications**	135 (1.6)	81 (1.9)	0.283
**Hyopthyroidism**	303 (3.7)	235 (5.6)	<0.001
**Renal failure**	585 (7.1)	362 (8.6)	0.003
**Liver disease**	369 (4.5)	193 (4.6)	0.832
**Peptic ulcer disease**	10 (0.1)	4 (0.1)	0.893
**AIDS**	19 (0.2)	11 (0.3)	0.893
**Lymphoma**	80 (1.0)	33 (0.8)	0.344
**Metastatic cancer**	502 (6.1)	256 (6.1)	1.000
**Solid tumor without metastisis**	867 (10.5)	397 (9.4)	0.058
**Rheumatoid arthritis/collagen vascular disease**	73 (0.9)	44 (1.0)	0.443
**Coagulopathy**	632 (7.7)	651 (15.4)	<0.001
**Obesity**	190 (2.3)	132 (3.1)	0.007
**Weight loss**	172 (2.1)	97 (2.3)	0.476
**Fluid and electrolyte disorders**	1329 (16.1)	946 (22.4)	<0.001
**Blood loss anemia**	6 (0.1)	7 (0.2)	0.218
**Deficiency anemia**	33 (0.4)	18 (0.4)	0.943
**Alcohol abuse**	186 (2.3)	95 (2.3)	1.000
**Drug abuse**	49 (0.6)	30 (0.7)	0.509
**Psychosis**	30 (0.4)	18 (0.4)	0.701
**Depression**	93 (1.1)	44 (1.0)	0.736

Data are presented as n (%).

Table S2. All Admission diagnoses with n > 50

ICD-10	Description	Norepinephrine	No norepinephrine	p-value
**S06**	Intracranial injury	178 (4.22)	357 (4.33)	0.81
**J80**	Adult respiratory distress syndrome	180 (4.27)	263 (3.19)	0.0026
**J12**	Viral pneumonia, not elsewhere classified	146 (3.46)	280 (3.4)	0.9
**I63**	Cerebral infarction	152 (3.6)	217 (2.63)	0.003
**I61**	Intracerebral hemorrhage	107 (2.54)	218 (2.65)	0.76
**J96**	Respiratory failure, not elsewhere classified	55 (1.3)	269 (3.27)	<0.001
**I21**	Acute myocardial infarction	105 (2.49)	192 (2.33)	0.62
**A41**	Other sepsis	86 (2.04)	176 (2.14)	0.77
**I46**	Cardiac arrest	44 (1.04)	193 (2.34)	<0.001
**J18**	Pneumonia, unspecified organism	34 (0.81)	195 (2.37)	<0.001
**I25**	Chronic ischemic heart disease	200 (4.74)	18 (0.22)	<0.001
**I35**	Nonrheumatic aortic valve disorders	167 (3.96)	35 (0.42)	<0.001
**C56**	Malignant neoplasm of ovary	72 (1.71)	103 (1.25)	0.049
**C78**	Secondary malignant neoplasm of resp and digestive organs	39 (0.92)	131 (1.59)	0.003
**R40**	Somnolence, stupor and coma	26 (0.62)	133 (1.61)	<0.001
**I71**	Aortic aneurysm and dissection	77 (1.83)	81 (0.98)	<0.001
**S72**	Fracture of femur	66 (1.57)	89 (1.08)	0.026
**I60**	Subarachnoid hemorrhage	70 (1.66)	81 (0.98)	0.002
**D37**	Neoplasm of uncrt behavior of oral cavity and dgstv organs	46 (1.09)	96 (1.17)	0.78
**C16**	Malignant neoplasm of stomach	28 (0.66)	106 (1.29)	0.002
**J44**	Other chronic obstructive pulmonary disease	23 (0.55)	111 (1.35)	<0.001
**C25**	Malignant neoplasm of pancreas	33 (0.78)	92 (1.12)	0.094
**E87**	Other disorders of fluid, electrolyte and acid-base balance	8 (0.19)	116 (1.41)	<0.001
**N18**	Chronic kidney disease (CKD)	29 (0.69)	86 (1.04)	0.062
**T07**	Unspecified multiple injuries	33 (0.78)	82 (1)	0.28
**C15**	Malignant neoplasm of esophagus	23 (0.55)	90 (1.09)	0.003
**C22**	Malignant neoplasm of liver and intrahepatic bile ducts	20 (0.47)	91 (1.1)	<0.001
**N17**	Acute renal failure	28 (0.66)	82 (1)	0.077
**D43**	Neoplasm of uncertain behavior of brain and central nervous system	49 (1.16)	57 (0.69)	0.010
**K92**	Other diseases of digestive system	23 (0.55)	81 (0.98)	0.015

**G40**	Epilepsy	18 (0.43)	81 (0.98)	0.001
**C34**	Malignant neoplasm of bronchus and lung	40 (0.95)	58 (0.7)	0.18
**R10**	Abdominal and pelvic pain	41 (0.97)	57 (0.69)	0.12
**I50**	Heart failure	24 (0.57)	71 (0.86)	0.095
**T84**	Complications of internal orthopedic prosthetic devices, implants and grafts	36 (0.85)	58 (0.7)	0.42
**K56**	Paralytic ileus and intestinal obstruction without hernia	42 (1)	51 (0.62)	0.028
**R06**	Dyspnoea	30 (0.71)	58 (0.7)	1
**I62**	Other and unspecified nontraumatic intracranial hemorrhage	24 (0.57)	62 (0.75)	0.29
**C79**	Secondary malignant neoplasm of other and unspecified sites	28 (0.66)	49 (0.59)	0.73
**I65**	Occlusion and stenosis of precerebral arteries, not resulting in cerebral infarction	22 (0.52)	55 (0.67)	0.39
**I67**	Other cerebrovascular diseases	16 (0.38)	55 (0.67)	0.058
**R57**	Shock, not elsewhere classified	19 (0.45)	50 (0.61)	0.32
**C18**	Malignant neoplasm of colon	26 (0.62)	42 (0.51)	0.52
**K72**	Hepatic failure, not elsewhere classified	17 (0.4)	49 (0.59)	0.21
**D38**	Neoplasm of uncertain or unknown behaviour of middle ear and respiratory and intrathoracic organs	31 (0.74)	30 (0.36)	0.008
**I26**	Pulmonary embolism	8 (0.19)	53 (0.64)	<0.001
**S32**	Fracture of lumbar spine and pelvis	27 (0.64)	34 (0.41)	0.11
**I70**	Atherosclerosis	18 (0.43)	42 (0.51)	0.62
**K70**	Alcoholic liver disease	7 (0.17)	53 (0.64)	<0.001
**C20**	Malignant neoplasm of rectum	18 (0.43)	40 (0.49)	0.75
**I74**	Arterial embolism and thrombosis	21 (0.5)	35 (0.42)	0.66
**M48**	Other spondylopathies	23 (0.55)	30 (0.36)	0.19
**I34**	Nonrheumatic mitral valve disorders	36 (0.85)	16 (0.19)	<0.001
**K83**	Other diseases of biliary tract	16 (0.38)	36 (0.44)	0.75
**G41**	Status epilepticus	23 (0.55)	61 (0.74)	0.25

Data are presented as n (%).
